# Fatty Acids and their Proteins in Adipose Tissue Inflammation

**DOI:** 10.1007/s12013-023-01185-6

**Published:** 2023-10-04

**Authors:** Rahul Mallick, Sanjay Basak, Ranjit K. Das, Antara Banerjee, Sujay Paul, Surajit Pathak, Asim K. Duttaroy

**Affiliations:** 1 A.I. Virtanen Institute for Molecular Sciences, University of Eastern Finland, Kuopio, Finland; 2 Molecular Biology Division, ICMR-National Institute of Nutrition, Indian Council of Medical Research, Hyderabad, India; 3 Department of Health and Biomedical Sciences, University of Texas Rio Grande Valley, Brownsville, TX USA; 4 Chettinad Academy of Research and Education (CARE), Chettinad Hospital and Research Institute (CHRI), Department of Medical Biotechnology, Faculty of Allied Health Sciences, Chennai, India; 5 Tecnologico de Monterrey, School of Engineering and Sciences, Campus Queretaro, Av. Epigmenio Gonzalez, No. 500 Fracc, San Pablo, Queretaro 76130 Mexico; 6 Department of Nutrition, Institute of Basic Medical Sciences, Faculty of Medicine, University of Oslo, POB 1046 Blindern, Oslo, Norway

**Keywords:** Metabolic syndrome, Dietary fats, n-3 PUFA, LCPUFA, Obesity, Inflammation

## Abstract

Chronic low-grade adipose tissue inflammation is associated with metabolic disorders. Inflammation results from the intertwined cross-talks of pro-inflammatory and anti-inflammatory pathways in the immune response of adipose tissue. In addition, adipose FABP4 levels and lipid droplet proteins are involved in systemic and tissue inflammation. Dysregulated adipocytes help infiltrate immune cells derived from bone marrow responsible for producing cytokines and chemokines. When adipose tissue expands in excess, adipocyte exhibits increased secretion of adipokines and is implicated in metabolic disturbances due to the release of free fatty acids. This review presents an emerging concept in adipose tissue fat metabolism, fatty acid handling and binding proteins, and lipid droplet proteins and their involvement in inflammatory disorders.

## Introduction

Adipose tissue contains multiple cell types, such as adipocytes, monocytes/macrophages, pericytes, endothelial cells, and stem cells. Adipose tissue is a loose connective dynamic tissue with many functions [1]. Adipose tissue stores triacylglycerol (TAGs) and regulates the secretion of free fatty acids (FFAs) to the plasma for their transport and metabolism in different tissues. Promoting lipolysis and releasing FFAs into the bloodstream also affects adipose tissue metabolism [2]. Adipose tissue fatty acid uptake involves several steps, including intestinal absorption, incorporation into chylomicron TAGs, and the subsequent release of FFAs by hydrolysis of TAGs by lipoprotein lipase (LPL) for adipocyte uptake. After being released from adipose tissue, FFAs are transported into circulation for delivery to various tissues.

Adipose tissue is increasingly considered an essential connector of cardiovascular disease (CVD), diabetes mellitus and insulin resistance, inflammation, and other obesity-related disorders. Adipose tissue regulates whole-body metabolism by altering the function of the liver, brain, heart, skeletal muscle, and vascular endothelium via the secretion of adipokines, resistin, leptin, fatty acid binding protein (FABP), and other factors. Adipose tissue has two types: brown adipose tissue (BAT) and white adipose tissue (WAT) [3]. WAT is distributed in several depots in the body, including subcutaneous, visceral, and other organs. Subcutaneous and visceral adipocytes are physiologically different from each other. WATs are less insulin sensitive, critical for energy storage, metabolically active, and have greater lipolytic activity [4].

**Fig. 1:**
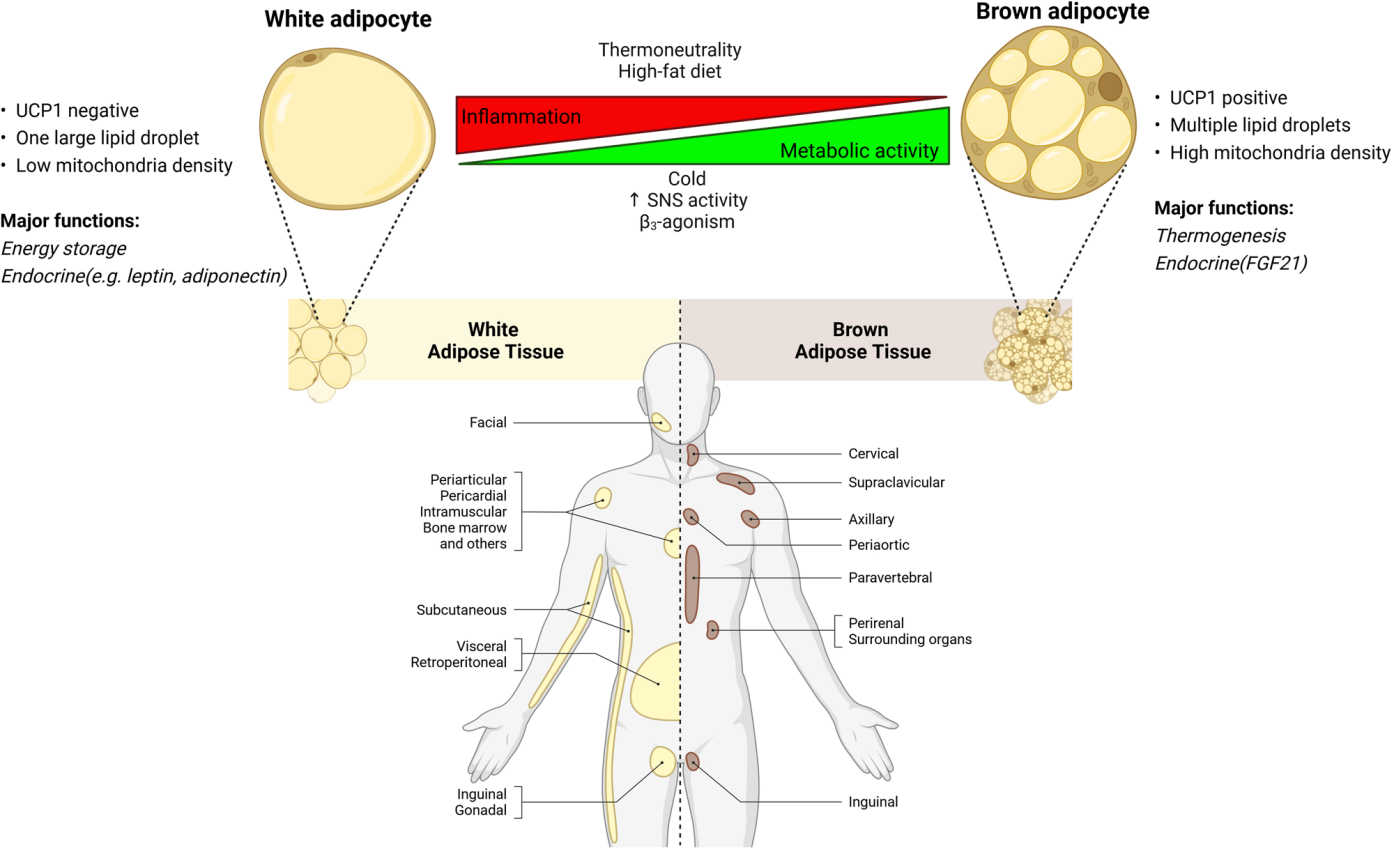
Adipose tissue types, location, and browning process. White adipocytes are large unilocular droplets enriched with a small number of small mitochondria, with a little UCP1 expression. In contrast, brown adipocytes are small multilocular with dense mitochondria with a high UCP1 expression. White adipose tissue is predominant in amount, supporting energy storage, and metabolically less active. On the other hand, brown adipose tissue is metabolically active, produces heat energy, promotes thermogenesis and browning (beige adipocytes) and reduces inflammation. White adipocytes can convert into brown adipocytes via cold stimulus, sympathetic nervous system (SNS) overdrive, and β3-agonism. UCP1, uncoupling protein 1

On the other hand, BAT produces thermogenesis from TAGs in humans and is present mainly in newborns. BAT was initially thought to maintain body temperature in human babies, but imaging studies confirmed metabolically active BAT in thoracic parts of adult humans [5]. BAT is involved in fat oxidation, thus maintaining the physiological function of adipose tissue in humans. Despite recent studies on the significance of BAT, its deposits do not appear to have a substantial impact on adult human metabolism [6]. Another adipose subtype, “beige,” is recently reported. Figure 1 describes the adipose tissue types, location, and browning process. Beige adipose contains brown and white adipocytes that develop within subcutaneous WAT. Activated beige adipose tissue can enhance body weight loss and inhibits obesity [7]. Thus, adipose is vital for metabolic homeostasis, whereas its dysfunction contributes to obesity and associated metabolic disease.

Obesity, metabolic syndrome, CVD, diabetes mellitus, and insulin resistance are public health challenges worldwide [8]. A crucial pathophysiological basis of obesity involves excess adiposity, associated with hypertrophy and hyperplasia due to the storage of excess fats [9]. Visceral adiposity is associated with a higher risk of obesity-related disorders such as insulin resistance, type 2 diabetes, CVD, and dyslipidemia [10] than peripheral adiposity, as per epidemiological observation. A relationship between inflammation and peripheral lipids is observed in the metabolic syndrome [11–13]. Dietary fats and their metabolism can affect inflammation by synthesizing various lipid mediators, immune response, and homeostasis [14, 15]. Adipose tissue can regulate inflammation by producing fatty acid-binding proteins, adipokines, resistin, leptin, and lipid droplet proteins [16, 17]. This review describes the roles of fatty acids and their binding proteins in adipose tissue and lipid droplets in modulating obesity-linked inflammatory diseases.

## Fatty Acid Uptake and Metabolism in Adipose Tissue

The effect of fatty acids on adipose tissue metabolisms and consequently on vascular functionality, inflammatory response, blood pressure, and hemostasis are reviewed [18]. Adipose tissue is critically involved in the uptake, storage, and metabolism of fatty acids. Blood fatty acids can be taken up into adipose tissue, either stored as TAGs or oxidized to provide energy [19]. Insulin supports the uptake of fatty acids as well as glucose into the adipose tissue [20]. Besides insulin, growth hormones, glucocorticoids, and catecholamines can control the uptake and metabolism of fatty acids in this tissue [21]. After being stored in adipose, fatty acids are processed in one of two ways: lipogenesis or lipolysis. As opposed to lipolysis, which breaks down stored triglycerides into fatty acids to produce energy, lipogenesis is the process by which fatty acids are synthesized and stored in TAGs [22]. A complicated interaction of hormones and metabolic cues controls the ratio of lipogenesis to lipolysis [22]. Lipid droplets in adipose tissue comprise lipid esters produced by the adipocytes or from circulating lipids [23]. Adipocytes release FFAs into the bloodstream via lipolysis of lipids from lipid droplets involving several lipases such as adipose triglyceride lipase (ATGL), hormone-sensitive lipase (HSL), and monoglyceride lipase (MGL) [24]. Several natriuretic peptides, insulin, and catecholamines regulate the activity of HSL and ATGL. Basal lipolysis and insulin resistance correlate with high TAG levels [25].

Adipocytes regulate plasma lipid profile via the uptake of fatty acids from TAGs of circulating lipoprotein particles, such as chylomicrons and VLDLs [26]. Since adipocytes are a great source of LPL, dysfunction in the TAG/LPL axis can adversely affect plasma lipids-associated disorders [27]. After hydrolysis, FFAs are taken up by the adipocytes, and they are re-esterified in the TAGs [28]. Fatty acids can be differentially incorporated into chylomicrons or hydrolyzed from chylomicrons. For example, eicosapentaenoic acid,20:5n-3 (EPA) esters in chylomicrons, are relatively resistant to hydrolysis by LPL compared to other polyunsaturated fatty acids (8). Consequently, EPA may accumulate at the surface of chylomicron remnants as TAG or DAG and are not immediately available for tissue uptake.

**Fig. 2:**
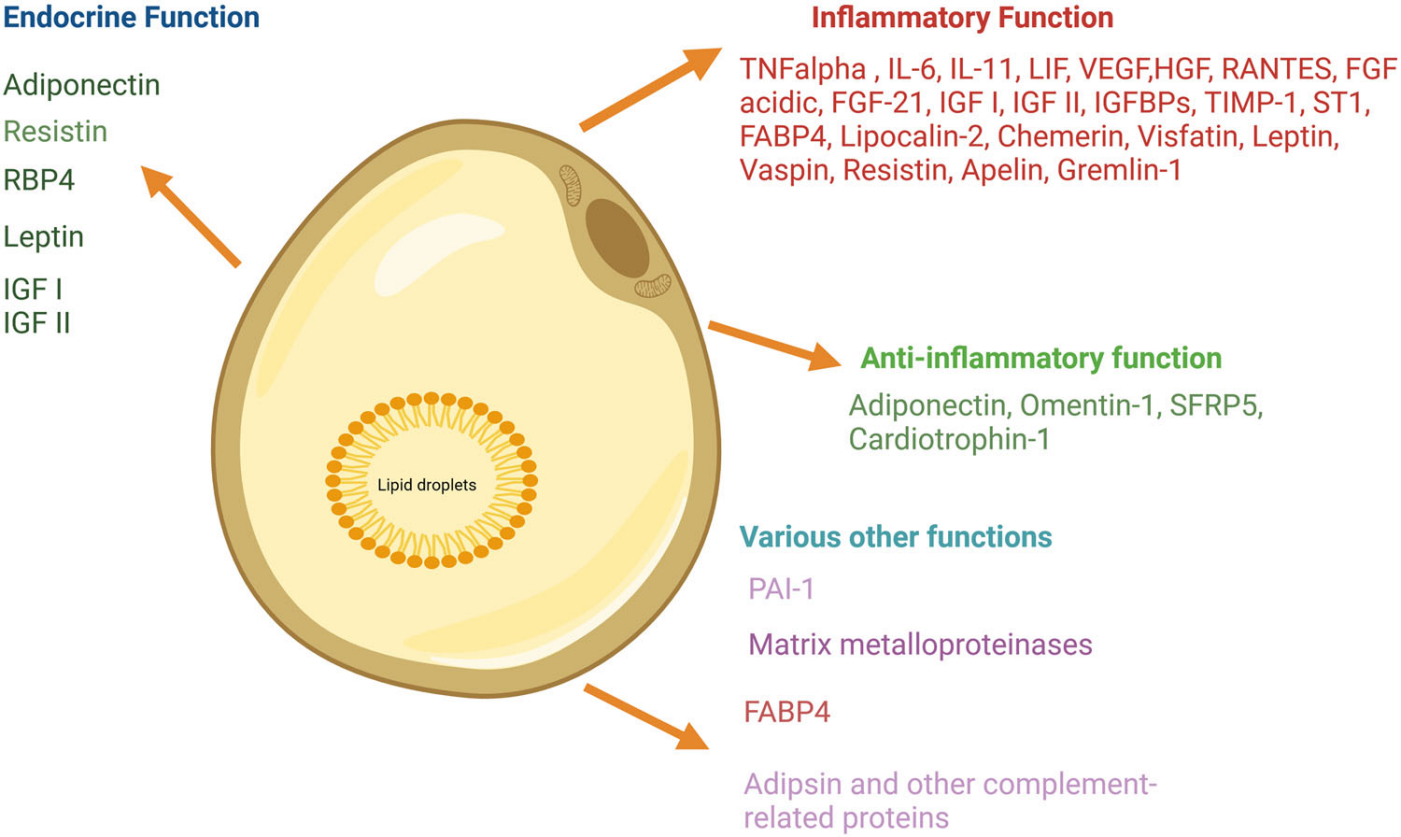
Modulation of inflammation by adipose tissue types. White adipose tissue predominantly secretes adipokines from adipocytes in lean subjects, while pro-inflammatory cytokines are secreted from macrophages, neutrophils, and activated T-cells in adipose tissue in obese persons. Adipose tissue in lean supports angiogenesis, insulin sensitivity, and anti-inflammation, while adipose tissue dysfunction is reported in obese subjects. IFN interferon, IL interleukin, TNF tumor necrotic factor, FABP4 fatty acid binding protein4, Th1 type 1 T helper cells, MCP-1 monocyte chemoattractant protein-1

Adipose tissue can modulate other tissues’ insulin sensitivity and energy metabolism by secreting hormones and cytokines [29]. For example, adipose tissue can affect the hypothalamus to control hunger and energy by producing leptin [30]. Adiponectin, which improves insulin sensitivity and has anti-inflammatory properties, is also produced by adipose tissue [31]. Normally, adipose tissue stores lipids during the postprandial state, whereas it releases fatty acids to provide energy in an energy-demanding state. Metabolic disorders observed in obesity, insulin resistance, and type 2 diabetes can be attributed to dysregulated adipose tissue metabolism and hormone/adipokines secretion [18, 32]. Figure 2 describes the involvement of adipose tissue types in modulating inflammation.

The adipocyte hypertrophy and increased basal lipolysis could result in adipose dysfunction in obesity-linked diseases [33]. Thus, understanding the factors that regulate adipose lipid droplets may reveal the mechanisms of obesity-associated risks in metabolic disorders.

## The Transport of Fatty Acids: Roles of Adipose Tissue Proteins

Circulatory fatty acids are transported to adipose tissue as FFAs. Apart from simple diffusion, fatty acid transport into adipocytes is a protein-mediated process. Membrane transport and intracellular transport of FFAs are mediated via several membrane fatty acid binding/transport (FAT/CD36, FATPs, and FABPpm) and intracellular FABPs [34, 35]. These proteins are involved with both the cellular uptake and the intracellular transport of long-chain fatty acids (LCFAs) (c > 14) [36]. Intracellular transport of LCFAs requires the involvement of low molecular weight proteins (15 kDa) known as cytoplasmic fatty acid-binding proteins (FABPs) [37]. Fatty acid membrane transport and fatty acid-binding proteins greatly influence the uptake and metabolism of fatty acids in adipose tissue.

The presence of FABP in rat jejunum was first reported by Ockner et al. in 1972 [38]. Subsequently, several FABPs were detected in different tissues, such as the liver, myocardium, adipose tissue, placenta, brain, kidney, and prostate [34]. FABPs are named after the first tissue from which they are detected [39]. In mammals, nine different FABPs with a tissue‐specific distribution were identified. [40]. However, the contribution of different FABPs and their dysregulation to the progression of disorders associated with various diseases still needs to be fully understood [41, 42]. FABPs are thought to be involved in high-fat diet-mediated obesity, diabetes, dyslipidemia, atherosclerosis, and modulation of immune responses [43]. Therefore, targeting FABPs associated with metabolism, cell growth, and proliferation may offer therapeutic opportunities for various pathologies in metabolic diseases. FABPs are highlighted as potential therapeutic targets for various related disorders, including obesity, diabetes, and atherosclerosis.

Membrane protein transporters that facilitate adipose fatty acid uptake are fatty acid translocase (FAT/CD36), plasma membrane fatty acid-binding protein (FABPpm), and fatty acid transport protein (FATP4) [35, 37, 44, 45].

Adipose FABP or FABP4 is abundantly expressed in adipose tissue, and several elements, including FFAs, insulin and glucocorticoids, and others, control its expression [46]. Abnormal expression or dysfunction of these proteins is involved in several diseases, especially those concerning lipid metabolism. FAT/CD36 was identified as a critical LCFA transporter in the adipose tissue [47]. Expression of FABPpm is increased during the preadipocyte differentiation [34]. FATP4 affects adipose droplet size and pool size of other complex lipids. Once inside, FFAs are bound by FABP, thus stimulating fatty acid absorption and cytoplasmic transportation [37]. FABP4 is the most abundantly expressed in adipocytes though the relative content of FABP4 in different fat tissue depots in humans. FABP4 involves in the intracellular traffic of FFAs and their subsequent metabolism.

The FABP4 mRNA level is co-related with the insulin levels in obese individuals [48]. Expression of FABP4 in adipose tissue and macrophages is associated with inflammation [49]. However, no data is available on the expression of FABPs/FATPs/CD36/ FABPpm involved in the accretion of lipids in the visceral and subcutaneous adipose of morbidly obese. Although cellular trafficking of FABP in modulating various metabolic diseases have been proposed [50], limited data are available on the direct roles of FABP4 in adipose tissue inflammation in human. The FABP4’s presence in macrophages has been linked with the promotion of inflammation in adipocytes [51], and coronary atherosclerosis [52] in modulating CVD risks.

The adipose depot acts as a buffer for the high consumption of dietary fats. Therefore, fatty acid protein transporters’ expression and/or content would be enhanced to compensate for the increased availability of lipids. Aberrant circulatory FABP4 level is associated with insulin resistance [52], diabetes mellitus [53], gestational diabetes, and metabolic syndrome [54]. FABP4 is related to atherosclerosis and CVD prevalence [52, 55]. The amount of FABP4 mRNA is higher in the epicardial than in the subcutaneous adipose. FABP4 mRNA is associated with atherosclerosis status in patients who underwent coronary bypass surgery [55]. The FABP4 levels also correlate to the extent of left atrial adipose tissue volume in patients with atrial fibrillation. FABP4 is a marker of atrial fibrillation after ablation [56]. Expression of FABP4 and inflammation genes was inhibited by the sodium-glucose cotransporter 2 inhibitor [57], suggesting its possible relationship with hypertension and inflammation. FABP4 can synergistically increase fatty acid oxidation with leptin’s help during adipose inflammation. However, their effects on mitochondrial fatty acid oxidation remain unclear.

Metabolic syndrome with combined features of central obesity with insulin resistance, dyslipidemia, and hypertension increases the ĆVD risk [58]. The dietary fat types can affect insulin sensitivity via several lipid mediators mechanisms, changing cell membranes’ fatty acid composition and others [59]. Dietary fat rich in unsaturated fatty acids, such as the Mediterranean diet, may prevent the development of metabolic syndrome, diabetes, and CVD risk [60–63]. However, the detrimental effect of consuming saturated fat or the benefit of polyunsaturated fat in humans is controversial [64–68].

On the other hand, a saturated fat-containing diet promotes obesity, insulin resistance, and metabolic syndrome [64, 69, 70]. Furthermore, the roles of fatty acids and their metabolites in developing chronic, low-grade inflammatory states in metabolic syndrome are elucidated [71]. However, a recent meta-analysis showed that a pro-inflammatory diet was not associated with an increased risk of metabolic syndrome. Still, it was significantly associated with a 35% higher risk of CVD [72]. Furthermore, a high-fat diet containing saturated fat increases the incidence of CVD by raising total plasma cholesterol and LDL cholesterol. However, a meta-analysis did not find evidence to associate high-fat intakes with an increased risk of CVD [73].

FAT/CD36 is involved in LCFA transport across the plasma membrane in many tissues, including the adipose tissue [47, 74–78]. The mRNA and protein expression of membrane fatty acid uptake/binding proteins (FAT/CD36, FABPpm, FATP4) in the visceral and subcutaneous adipose and the LPL, PPARγ, FABP4, and FABP5 of obese patients were investigated. An increase was observed in the expression of FAT/CD36 in obese patients. The increased expression of the FAT/CD36 gene is at least partly due to the excessive lipid in adipose. Increased expression of FAT/CD36 at mRNA and plasma membranes protein levels) in visceral adipose in patients with morbid obesity. Bower et al. [79] demonstrated that the mRNA expression of FAT/CD36 in the visceral adipose of obese Afro-American and Caucasian women is higher than in Caucasian women. In addition to the observation in morbidly obese Caucasian subjects, an elevated plasma membrane FAT/CD36 and mRNA expression were also measured in the visceral adipose of lean individuals. However, the visceral adipose of obese subjects might have more potential for the uptake of LCFA, similar to other reports [79].

An increased expression of both PPARγ and LPL was observed in adipose tissue in obese individuals. PPARγ, the promoter of adipogenesis, acts as a transcription factor for the expression of FAT/CD36 [80]. Thus, increased LPL expression and FAT/CD36 in adipose increased the storage of TAGs in adipose. FABPpm mRNA expression was substantially lower in the subcutaneous and visceral adipose obese individuals compared to the non-obese subjects. Lappas et al. [81] also demonstrated a significant decrease in FABPpm mRNA expression in the subcutaneous adipose of obese people. However, the decrease in mRNA levels did not correlate with FABPpm protein levels. No change in FATP4 mRNA and protein expression in the subcutaneous and visceral adipose of the subjects with obesity compared to the lean control. Thus, FATP4 may not directly affect fatty acid uptake by adipocytes. This was also demonstrated in a mouse model with inactivated adipocyte f*atp4* gene [82]. This study showed that the lack of FATP4 expression did not affect fatty acid uptake in these cells. Several studies demonstrated that FAT/CD36 is a major LCFA transporter for adipose fatty acid uptake, especially in the visceral adipose tissue [47].

## Fatty Acid Storage in Adipose: Roles of Lipid Droplet Proteins

Lipid droplets are the organelles that store lipids in adipose tissue. The size and composition of lipid droplets depend on cellular metabolic status and environmental factors. Lipid droplets are predominantly composed of TAGs [83]. Fatty acids released from lipid droplets act as signaling molecules or precursors for bioactive lipids, including eicosanoids, retinoic acid, endocannabinoids, and ceramides [84]. Adipose tissue takes up fatty acids and stores them in lipid droplets via different fatty acid membranes and cellular transporters, and binding proteins. Emerging studies have revealed that lipid droplets protect cellular integrity and function via other mechanisms. For example, lipid droplet-mediated signaling affects mitochondrial function, the lipid metabolism [85], and the inflammation [84, 86]. Lipid droplets can consume excess lipids, protecting cells from lipotoxicity. Excessive lipid accumulation in lipid droplets can lead to obesity, diabetes, atherosclerosis, and fatty liver [87–89]. Similarly, a lack of lipids or lipid droplets in adipose tissues can lead to diseases such as lipodystrophy.

The dysregulation of lipid droplets can lead to disease states. Lipid droplets protect cell membranes and maintain cell homeostasis, modulate autophagy, provide signaling mediators, sequester toxic lipids and proteins, store energy, and preserve the redox balance [90, 91]. Lipid droplets carry out these functions in cooperation with other cellular organelles. However, the complex interplay between lipid droplets and other organelles is still unknown.

Several proteins are required for regulating lipid storage in lipids droplets. Lipid droplet structural proteins maintain lipid droplets’ function, structure, and morphology. Perilipin 1 (PLIN1) is the most abundant lipid droplet that protects lipid droplets from lipolysis. PLIN1, ADRP/ADFP/PLIN2, Tip47/PLIN3, S3-12/PLIN4, and OXPAT/PLIN5, the so-called “PAT” family proteins, are the main structural proteins are renamed as PLIN1-5 [92]. PLIN1 and PLIN2 are predominant subtypes localized in the lipid droplets [93]. PLIN1 regulates the accessibility of lipases to lipid droplets [94]. PLIN1 regulates lipid storage and lipolysis in response to the metabolic requirement of the adipose tissue. PLIN1 is phosphorylated by cAMP-dependent protein kinase A (PKA). The phosphorylated form of PLIN1 increases lipid storage or lipolysis depending on the metabolic status of the cell [94]. PLIN2 is predominantly present in the liver and muscle and involved in hepatic lipid accumulation [95], and improves insulin sensitivity in the skeletal muscle [96]. Like PLIN2, PLIN5 increases TAG storage and suppresses insulin resistance [96]. Additionally, COX-2 is found in inflamed lipid droplets, and the product of this enzyme carries essential inflammatory signals [97, 98]. Lipid droplets containing MAPKs, PKC, PI3K, and other critical enzymes are implicated in the intracellular signaling of diverse cellular responses [99, 100].

## Adipose Tissue-derived Proteins and their Roles in Inflammation

**Fig. 3:**
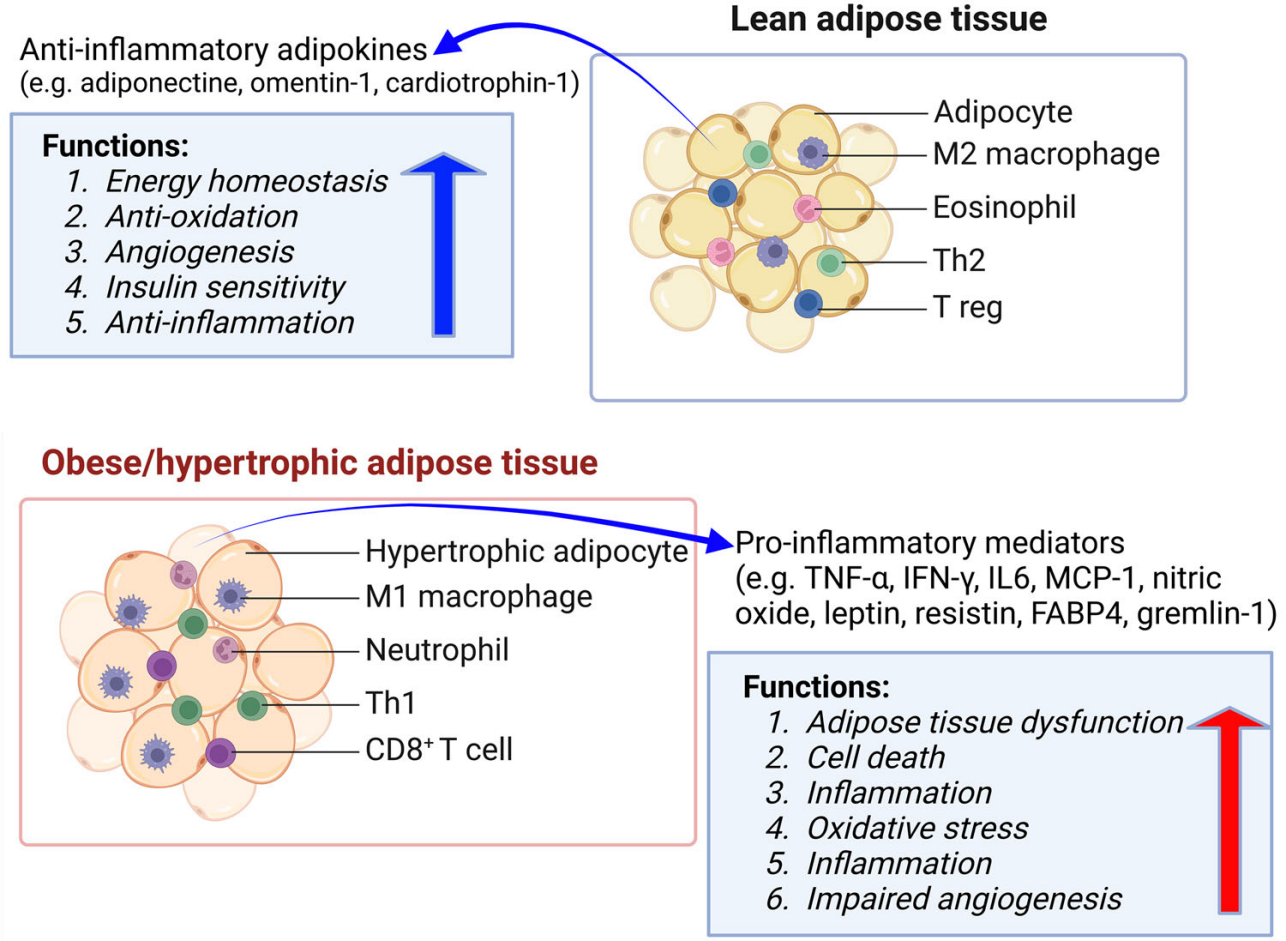
Adipose tissue secreted proteins and their functions. Adipocyte primarily stores energy through triacylglycerols and secretes many proteins involved in endocrine and inflammation modulatory functions. RBP4: retinal binding protein 4, IGF: insulin growth factors, IL: interleukin, TNF tumor necrotic factor, LIF leukemia inhibitory factor, VEGF vascular endothelial growth factor, HGF hepatocyte growth factor, RANTES regulated on activation, normal T cell expressed and secreted, FGF fibroblast growth factor, IGFBP insulin growth factor binding protein, TIMP1 tissue inhibitor of metalloproteinases, FABP4 fatty acid binding protein 4

The adipose depot secretes various proteins such as cytokines, chemokines, and hormonal factors that regulate inflammation, immunity, adiposity, and lipid metabolism. Adipokines are released and synthesized by adipose tissue and are crucial for controlling inflammation and immune system function. More than 600 adipokines have been identified [101]. These adipokines and fatty acids maintain metabolic homeostasis through the adipose tissue cross-talk with other tissues [102]. The adipose tissue performs as an endocrine organ and secreted several proteins those potentially modulate inflammatory function in target tissues. The dysregulation of these proteins leads to several metabolic and inflammatory diseases. Figure 3 describes adipose tissue-secreted proteins and their inflammation modulatory functions.

FFAs produced by adipose lipolysis can act as inflammatory mediators in immune cells. ATGL-mediated lipolysis regulates the synthesis of pro-inflammatory eicosanoids in mastocytes [103]. ATGL-mediated lipolysis increased lipid droplets but reduced the levels of eicosanoids in human-activated mastocytes. This suggests that lipolysis of TAGs produces arachidonic acid,20:4n-6, a precursor for eicosanoids [103]. Similarly, inhibition of ATGL caused a significant decrease of PGD_2_, LTE_4_, LTB_2_, and thromboxane B_2_ in murine neutrophils. Exudates from ATGL-deficient mice significantly inhibited eicosanoid synthesis, indicating that this is involved in inflammatory signaling processes [84]. Inhibition of HSL reduced lipolysis by the non-selective β-adrenergic agonist-mediated release of eicosanoids. Lean WAT has regulatory and immunosuppressive immune cells such as M2-like adipose macrophages, regulatory T cells, Th2 cells, iNKT cells, and eosinophils. The M2 adipose macrophages are uniformly distributed within the tissue and perform various physiological functions, inhibiting the proliferation of adipocyte progenitors and secreting anti-inflammatory cytokines such as IL-10, IL-4, IL-13, and IL-1Rα [104, 105].

Leptin is the product of the *obese* (*ob*) gene in mice and the *lep* gene in humans [106]. Leptin is produced by the subcutaneous WAT [107]. In addition to controlling energy balance, leptin is associated with the immune system and inflammation. The leptin receptors, such as long (ObRb) and short form (ObRa), are expressed by immune cells. The binding of leptin to ObRb stimulates the proliferation of clonal immune cells [108]. Leptin also affects both the innate and adaptive immune systems.

Monocytes and adipose macrophages express ObRa and ObRb, whereas dendritic cells express ObRb. Leptin stimulates the proliferation of monocytes and the expression of inflammatory cytokines such as TNFα and IL-6 [109]. It also expresses pro-inflammatory cytokines such as IL-1β, IL-6, and MCP-1 in the eosinophils [110]. Leptin also promotes neutrophil chemotaxis. The migration of neutrophils to the peritoneum in mice is also induced by leptin. These effects are mediated by inducing the production of TNFα and chemokines by monocytes and macrophages [111]. The surface expression of the adhesion molecules ICAM-1 and CD18 increases, while ICAM-3 and L-selectin are down-loaded in eosinophils by leptin.

Adiponectin, another well-reported adipokine, has anti-inflammatory and insulin-sensitizing properties. Adipocytes produce and secrete a protein called adiponectin, which has reportedly inhibited the synthesis of pro-inflammatory cytokines like TNFα and IL-6 [112]. Additionally, improving insulin sensitivity, adiponectin is thought to control lipid and glucose metabolism [113].

Resistin, visfatin, and omentin are other adipokines affecting individuals’ inflammation and modulation of immunity. Insulin resistance and metabolic diseases have been linked to resistin’s pro-inflammatory actions [114]. Conversely, visfatin reduces inflammation and improves insulin sensitivity [115]. Another adipokine with anti-inflammatory properties, omentin, regulates glucose metabolism [116].

Thus, several adipose tissue-derived proteins are directly or indirectly involved in modulating insulin sensitivity and inflammation in the target tissue by controlling lipid and glucose metabolism.

## Fatty Acid-binding Protein 4 of Adipose Tissue and its Role in Inflammation

FABP4 activation has been linked to atherosclerosis, coronary artery disease, and heart failure due to its involvement in angiogenesis [117, 118]. It has been established that FABPs are dysregulated in the CVD [119, 120]. FABP4 plays a role in developing metabolic syndrome through various pathways involving adipocytes and macrophages [121]. The adipocytes [52] and macrophages [122] regulate the levels of FABP4 in circulation. Fatty acids, PPARγ agonists, insulin, lipopolysaccharide, and oxLDL regulate the levels of FABP4 in adipocytes and macrophages [123]. Reduced lipolysis was reported in FABP4-knockout mice [124], indicating that FABP4 regulates lipolysis.

Apolipoprotein-E-deficient mice with FABP4 deficiency did not develop atherosclerosis from a high-cholesterol diet [120]. However, the lack of FABP4 can modulate insulin resistance and lipid metabolism, but the mechanism is unknown. At the same time, the physiological role of FABP4 in circulation is unknown but can be used as a biomarker for metabolic syndrome and CVD [54]. FABP4 modulates inflammation by changing cholesterol concentration in macrophages. The cholesterol-lowering statin suppresses FABP4 expression in macrophages [125]. Increased cholesterol efflux was described in macrophages with elevated PPARγ isolated from FABP4-deficient mice. This indicates macrophage FABP4 plays a role in foam-cell formation via PPARγ–liver X receptor-α (LXRα)–ATP-binding cassette A1 (ABCA1) pathway. Reduced cytokine production and pro-inflammatory mediators, such as TNFα and COX2, were observed in macrophages isolated from FABP4-deficient mice [126]. Upregulation of FABP4 was not observed in adipocytes of FABP5-deficient mice due to a higher level of FABP4 [127].

In macrophages, the FABP4/FABP5 ratio is identical; no compensatory expression of FABP5 was observed in FABP4-knockout mice [128]. Various in vivo phenotypes on FABP5 expression are relevant to the metabolic syndrome. Overexpression of FABP5 in adipose tissue increased the lipolysis [129] and decreased insulin sensitivity [127]. On the other hand, increased insulin sensitivity in adipocytes from FABP5-deficient mice was reported [127]. The macrophage-loaded adipose tissues are responsible for inflammatory response, insulin resistance, and CVD in obesity [130]. FABP4 and FABP5 in adipocytes and macrophages contribute to inflammatory and metabolic disorders [51].

**Fig. 4:**
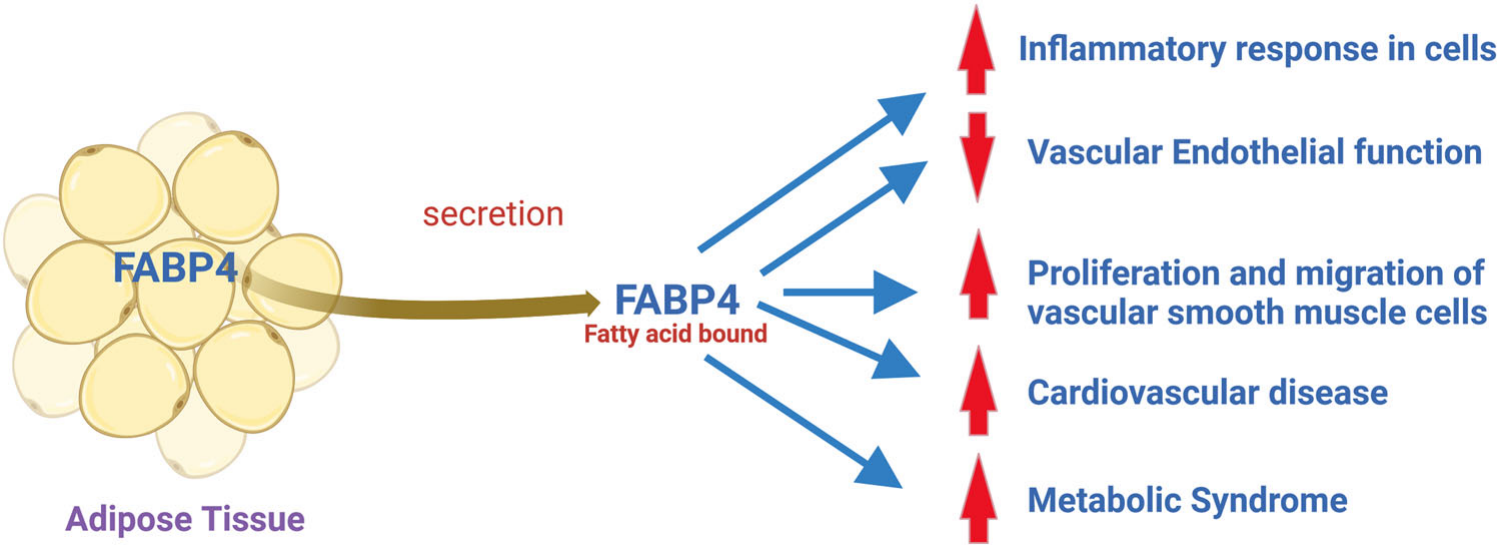
Putative mechanism of the development of CVD by secreted FABP4 from adipose tissue. The secretion of FABP4 from adipocytes can activate endothelial cells, increase the proliferation/migration of vascular smooth muscle cells (VSMC), and induce pro-inflammatory responses in macrophages, vascular endothelial cells, and VSMC, leading to the development of atherosclerotic phenotype

Adipocytes and macrophages were generally insulin-sensitive in FABP-deficient mice. Obese mice deficient in both FABP4 and FABP5 had reduced storage of fatty acids in adipose tissue and no insulin resistance [131]. ApoE^-/-^ mice had less atherosclerosis and increased survival compared to wild-type and individual FABP-knockout counterparts [132]. The mice deficient in FABP4 and/or FABP5 also exhibited increased plasma FFA levels [133]. This indicates that the bioavailability of intracellular FFAs is more relevant to developing metabolic syndrome. Increased plasma level of FABP4 in diabetic patients with peripheral arterial disease was described [119]. The enhanced plasma level of FABP4 was independent of age, sex, or prior history of CVD. Therefore it raises the possibility of using FABP4 plasma levels as a biomarker for diagnosing coronary artery disease risk in diabetic patients. FABPs are increasingly reported in modulating the pathophysiology of CVD, especially in inflammation and metabolic imbalance. However, more studies are required to understand how FABPs contribute to CVD pathogenesis and discover possible therapeutic targets for preventing and treating CVD. Figure 4 describes the putative mechanism of the development of CVD by secreted FABP4 from adipose tissue.

## Adipose Tissue Metabolism and Its Roles in Cardiovascular Disease

Adipose tissue is an important site of energy storage in the body and can be mobilized during energy demand through lipolysis. Lipolysis is regulated by a variety of angiogenic growth factors, including insulin, vascular endothelial growth factor (VEGF), and angiopoietin-like protein 4 (ANGPTL4)/ fasting-induced adipose factor (FIAF).

In adipose, insulin plays a crucial role in controlling lipolysis. Stimulating LPL, which converts TAGs into FFAs for storage, prevents lipolysis [134]. HSL, which converts TAGs into FFAs for use as fuel, is likewise inhibited by insulin [134]. Increased lipolysis and the release of excess FFAs can be caused by insulin resistance, a characteristic feature of obesity-associated type 2 diabetes. Insulin resistance can also promote inflammation and insulin resistance in sensitive target tissues, advancing the development of CVD [135]. Adipocytes produce VEGF, a growth factor that stimulates the development of angiogenesis. Obesity has been linked to higher VEGF levels and a higher risk of CVD [136].

Adipocytes produce the protein ANGPTL4, which is involved in controlling lipolysis. It blocks LPL, which decreases FFA absorption into adipose tissue and increases FFA release into the bloodstream [137]. Exercise and fasting raise ANGPTL4 levels, which have been linked to a lower risk of CVD [138]. ANGPTL4 regulates the catabolism of TAG-rich lipoproteins, thus controlling plasma levels of lipoproteins and the accretion of lipids in tissues. Though ANGPTL4 is relevant in whole-body lipid metabolism, the specific contribution of ANGPTL4 in regulating lipid metabolism in different tissues has yet to be discovered. In adipose tissue, the role of ANGPTL4 in ectopic lipid deposition [139], metabolism of lipids [140, 141], glucose homeostasis[137, 142], and vascular disease [143] are emerging.

Overall, the coordinated interplay of adipose lipolysis, insulin, VEGF, and ANGPTL4-mediated actions control the pathogenesis of developing CVD. Therefore, for optimal metabolic health and to lower the risk of CVD, adipose tissue must maintain a good balance between energy mobilization and storage.

## Dietary Fats and Their Cross-talks with Adipose Tissue: Effects on Inflammation and Metabolic Disease

Overall, the type and amount of dietary fats consumed can significantly affect adipose tissue inflammation, immune function, and the expression of adipose-derived proteins. Consuming a diet rich in unsaturated fats and low in saturated and trans fats may help to reduce inflammation and improve metabolic health. In obesity, inflammation in adipose tissue is one of the factors responsible for insulin resistance. Immune cells of both innate and adaptive immune systems in adipose tissue regulate inflammation and insulin resistance. M2 macrophages, eosinophils, and Tregs maintain insulin sensitivity in lean adipose tissue by secreting Th2-type cytokines. In contrast, M1 macrophages, Th1 cells, CD8 T cells, and mast cells that secrete Th1-type cytokines dominate adipose tissue via pro-inflammatory responses and insulin resistance in obese individuals. Adipocytes are critical regulatory cells that control inflammation through cytokine secretion and antigen presentation activity.

Dietary lipids contain saturated fatty acids (SFA), monounsaturated acids (MUFA), polyunsaturated fatty acids (PUFAs), and trans fatty acids. Therefore, the diet’s quality and amounts of fatty acids play a crucial role in several features of the CVD [144, 145]. Dietary lipids affect adipose tissue homeostasis by controlling the expression of adipose-derived proteins involved in immune response and inflammation. Furthermore, dietary lipids modulate the fatty acid composition of plasma membrane phospholipids [146, 147]. Adipose tissue inflammation caused by saturated and trans fats is linked to the release of inflammatory cytokines in immune cells [148]. The emergence of metabolic disorders, including type 2 diabetes mellitus and obesity, is assumed to be due to this persistent low-grade inflammation [149].

In contrast, diets high in n-3 and low n-6 PUFAs, have anti-inflammatory effects in adipose tissue [150]. These fats have been demonstrated to alter the adiponectin receptor in adipose tissue, which is linked to improved insulin sensitivity and anti-inflammatory properties [150]. Substituting dietary SFAs with MUFAs positively affects CVD risk factors. This exchange of fatty acids lowers LDL-cholesterol levels [151], improves the postprandial plasma lipid profile [152], and lowers blood pressure [14]. N-3 and n-6 PUFAs and their metabolites have differential metabolic effects on fat utilization in the body. The pro-inflammatory roles of excess n-6 PUFAs in promoting adipose tissue inflammation have recently been reviewed extensively [153]. Therefore, increased dietary intake of sea foods rich in n-3 LCPUFAs could be beneficial in improving several features of CVD risk factors. Docosahexaenoic acid, 22:6 n-3 (DHA), and EPA decrease levels of TAGs, increase plasma HDL-cholesterol, and have less pro-inflammatory effects, thus can inhibit CVD and metabolic syndrome.

Fatty acids are associated with the pathogenesis and treatment of the metabolic syndrome. Fatty acids are involved in membrane structure and function, energy, signaling, and immunoregulation. In addition, depending on the double bonds and chain length, fatty acids regulate the pathogenetic mechanisms of glucose transport disturbance, insulin resistance, chronic inflammation, oxidative stress formation, and mitochondrial dysfunction in MetS. For example, replacing SFAs with MUFAs produces beneficial effects on MetS.

N-3 PUFAs decreased body fat in mice compared with a low-fat diet, and SFAs or n-6 PUFA (equivalent energy) in rats fed for seven weeks on a high-fat diet [154]. The anti-obesity effects of n-3 PUFAs are mediated by WAT re-esterification, resulting in energy expenditure [155] and changing the fetal thermogenic development of adipose in mice [156]. The n-3 PUFA level in plasma was inversely correlated with insulin resistance and glucose intolerance in 447 Norton Sound Inuits [157]. Fish oil consumption decreased 40% with reduced oxidation of carbohydrates, increased lipids, and non-oxidative glucose disposal in healthy humans [158]. Fish oil consumption for two months to insulin-resistant rats increased the plasma level of adiponectin and reduced insulin resistance and dyslipidemia [159]. Feeding mice with partially replaced vegetable fats with EPA and DHA increased plasma adiponectin levels [160]. EPA enhanced the release of adiponectin in obese and high-fat diet-induced obese mice and obese people [158]. n-3 PUFAs may benefit metabolic syndrome via decreasing plasma TAG levels and adiposity and increasing plasma adiponectin levels. The n-3 PUFA-derived resolvins, protectins, and maresins are known as specialized pro-resolving lipid mediators (SPMs), autacoids proposed to mediate immuno-resolving activity due to their anti-inflammatory roles in reducing adipose inflammation, and enhances insulin sensitivity in rodent [161]. The inflammatory resolution is involved by reducing neutrophil infiltration and proinflammatory mediators, activating macrophage-mediated clearance, and tissue remodeling [162]. However, clinical findings about SPM’s roles in adipose inflammation are scanty.

Conjugated linoleic acids (CLAs) improve obesity and cardiovascular functions in several animal studies [163]. The effects of CLA on lipid metabolism depend on its isomers [164]. The anti-diabetic effects of CLA are due to the 10*t*,12*c*-isomer [165, 166]. A CLA mixture and the 10*t*,12*c*-CLA isomer prevented obesity-induced hypertension [167–169]. Many animal studies have demonstrated the anti-obesity, anti-atherogenic, anti-diabetic, and hypotensive effects of CLAs [170]. However, the mechanism of CLAs and isomer-specific impact on CVD risk factors in the clinical set-up is yet to be proved. CLAs increase the β-oxidation of fatty acids in adipose tissues and suppress fatty acid synthesis in hepatocytes. In addition to these effects, CLAs regulate the synthesis of adipokines, such as adiponectin, leptin, and angiotensinogen [167–169]. Consuming a CLA mixture (1.8 g/day) for three months decreased a 4% in body fat compared with olive oil in healthy men and women [171]. Supplementing conjugated DHA reduced the fat deposition in the liver and epididymal adipose tissue and improved lipid and carbohydrate metabolism in rats [172]. The anti-obesity and lipid-lowering effects of conjugated EPA were also demonstrated [173].

## Lipid Metabolism and Fatty Acid Binding Proteins are Involved in Inflammation-induced Endothelial Dysfunction

Adipose secretion of inflammatory IL-6, TNFα, and leptin contributes to endothelial dysfunction, platelet activation, hypercoagulability, and impaired fibrinolysis [174–177]. Plasma lipids interact with endothelial cells through specific cell membrane receptors or direct interchange with endothelial plasma membranes. LOX-1 (the receptor of oxLDL) is highly expressed when endothelial cells are incubated with oxLDL [90. Endothelial cells can recognize acetylated and oxLDL via surface receptors such as CD36/FAT and SR-B1 [178]. Lysophosphatidylcholine, an oxidative constituent of LDL, can reduce NO release from endothelial cells [179]. Other oxidative constituents of LDL, such as 13-hydroperoxyoctadecadienoate (13HPODE) and 7-ketocholesterol, and 7-β-hydroxycholesterol can affect the L-arginine-NO pathway in bovine aortic endothelial cells [179]. Endothelin-3 stimulated-NO release is suppressed by TAG-rich lipoproteins [180].

The endothelium is the source of some components essential for both thrombosis and fibrinolysis; definitive conclusions on the influences of lipids still need to be confirmed. Endothelial cells modulate coagulation and fibrinolysis processes. The effects of lipids on endothelium-dependent vasodilatation can be reversed by decreasing plasma lipids and providing L-arginine, the nitric oxide synthetase substrate, LDL and TAG-rich lipoproteins can stimulate the adhesion of leukocytes to the endothelial surface. Adipose tissue can control these processes via adipokines and lipid metabolism. Several cytokines, such as TNFα and IL-1, can induce adherence and penetration of the endothelium by leukocytes. The initial rolling represents an interaction between leukocytes and selectins, with subsequent adherence occurring through ICAM and VCAM. The expression of increased adhesion molecules for leukocyte adhesion was demonstrated in different conditions [181].

In contrast to elevated LDL’s pro-adhesive effects, HDL and apoA1 decrease the expression of adhesion molecules. P Selectin, stored in the Weibel-Palade bodies of endothelial cells, is expressed in the membranes of the atheromatous segments of human arteries. Hyperlipidemic and CVD patients show increased extracellular domains of adhesion molecules. Circulating ICAM and P selectin are increased in patients with CVD, and soluble ICAM, VCAM, and selectin in patients with hypertriglyceridemia [182].

## Conclusions

Dysfunctional adipose tissue is characterized by adipocyte hypertrophy, dyslipidemia, metabolic syndrome, and inflammation. Adipose tissue releases several bioactive factors, adipokines, and FABP4, which are involved in glucose and lipid metabolism. In obesity, some adipokines are upregulated and affect homeostasis via their pro-inflammatory, pro-atherosclerotic, or pro-diabetic properties. At the same time, beneficial adipokines are down-regulated and thus fail to protect normal adipose biology. A complex interaction of fatty acid-handling proteins, hormones, and adipokines controls the aspects of lipogenesis and lipolysis in adipose tissues. In addition to lipid droplet proteins, FABP4, FABPpm, and FAT/CD36 regulate adipose physiology and pathology. FABP4 is a critical lipid mediator of inflammation. Plsama levels of FABP4 are associated with metabolic syndrome and CVD. Due to its implication in various diseases, FABP4 has become a promising target for developing small molecule inhibitors and neutralizing antibodies for disease treatment. The genetic variation of these proteins may also play a role in obesity, metabolic syndrome, and the response to dietary and pharmacological therapies.
